# Correction format has a limited role when debunking misinformation

**DOI:** 10.1186/s41235-021-00346-6

**Published:** 2021-12-29

**Authors:** Briony Swire-Thompson, John Cook, Lucy H. Butler, Jasmyne A. Sanderson, Stephan Lewandowsky, Ullrich K. H. Ecker

**Affiliations:** 1 Network Science Institute, Northeastern University, Boston, USA; 2 The Institute for Quantitative Social Science, Harvard University, Cambridge, USA; 3 Monash University, Monash Climate Change Communication Research Hub, Melbourne, Australia; 4 Center for Climate Change Communication, George Mason University, Fairfax, VA USA; 5 School of Psychological Science, University of Western Australia, Perth, Australia; 6 School of Psychological Science, University of Bristol, Bristol, UK

**Keywords:** Belief updating, Misinformation, Continued influence effect, Corrections

## Abstract

Given that being misinformed can have negative ramifications, finding optimal corrective techniques has become a key focus of research. In recent years, several divergent correction formats have been proposed as superior based on distinct theoretical frameworks. However, these correction formats have not been compared in controlled settings, so the suggested superiority of each format remains speculative. Across four experiments, the current paper investigated how altering the format of corrections influences people’s subsequent reliance on misinformation. We examined whether myth-first, fact-first, fact-only, or myth-only correction formats were most effective, using a range of different materials and participant pools. Experiments 1 and 2 focused on climate change misconceptions; participants were Qualtrics online panel members and students taking part in a massive open online course, respectively. Experiments 3 and 4 used misconceptions from a diverse set of topics, with Amazon Mechanical Turk crowdworkers and university student participants. We found that the impact of a correction on beliefs and inferential reasoning was largely independent of the specific format used. The clearest evidence for any potential relative superiority emerged in Experiment 4, which found that the myth-first format was more effective at myth correction than the fact-first format after a delayed retention interval. However, in general it appeared that as long as the key ingredients of a correction were presented, format did not make a considerable difference. This suggests that simply providing corrective information, regardless of format, is far more important than how the correction is presented.

**Supplementary Information:**

The online version contains supplementary material available at 10.1186/s41235-021-00346-6.

## Significance statement

Misinformation is extremely prevalent, from misconceptions regarding climate change and vaccines to fallacies surrounding cancer and COVID-19. While several different formats have been proposed as superior, this has yet to be experimentally tested. For instance, some researchers propose that a “myth-first” format is best to correct misinformation; this is where a false claim is initially presented, followed by a false label and a subsequent explanation as to why the claim is false. By contrast, a “fact-first” approach—presenting the factual information prior to the misinformation—is often cited as preferable. Understanding why some correction formats are more effective than others can help tease apart various theoretical notions of *why* people continue to believe in misinformation and also has practical applications for fact-checkers. We conducted four experiments using a range of different materials that investigated how altering the format of corrections might influence people’s subsequent reliance on misinformation or induce sustained belief change. Our results indicate that correction format was not a strong determinant of belief change and that as long as the key ingredients of a correction were presented, format did not appear to make a considerable difference. This suggests that it may be more important for fact-checkers to focus on getting corrections (of any format) to the people most likely to hold relevant false beliefs, especially where such misconceptions have the greatest potential for harm.

## Introduction

Misinformation can continue to influence an individual’s memory and reasoning even after a clear correction has been elicited; a phenomenon known as the continued influence effect (Johnson & Seifert, 1994; Lewandowsky et al., 2012; Walter & Tukachinsky, 2020). Given that being misinformed can have negative ramifications on both the individual and society (e.g., Islam et al., 2020; Treen et al., 2020), finding optimal corrective techniques has become a key focus of research and educational campaigns (Walter et al., 2020). Understanding why some correction formats are more effective than others can also help tease apart various theoretical notions of *why* people continue to believe in or be influenced by corrected misinformation.

In the current paper we use the term “myth” to refer to a piece of real-world misinformation. One factor assumed to impact the effectiveness of myth corrections is the order in which various constituent parts of the correction are presented. At least three divergent correction formats (myth-first; fact-first; and fact-only) have been proposed as the superior corrective method based on distinct theoretical frameworks. However, these correction formats have not been compared in controlled settings, so the suggested superiority of each format remains speculative. To address this, we conducted four experiments using a range of different materials that investigated how altering correction format influences subsequent reliance on misinformation.

### Misinformation prior to correction: the myth-first approach

Traditionally, most fact-checking has used a myth-first format to disseminate corrective information. In this format, a false claim (the “myth”) is initially presented, followed by a false label, and a subsequent explanation as to why the claim is false (Guzzetti et al., 1993). This form of correction is often termed a *refutation* and has been found to be superior to a basic retraction that just labels a myth as false without providing factual details (e.g., see Ecker et al., 2010, 2020; Johnson & Seifert, 1994; Swire et al., 2017; Walter & Tukachinsky, 2020). Kendeou et al. (2014) suggested that the reason for the myth-first format’s relative success may be that activation of a misconception through initial presentation of the false claim may facilitate co-activation of misinformation and the correction—and associated conflict detection—when the correction is presented. Co-activation and conflict detection are thought to be conducive to knowledge revision (see also Ecker et al., 2017; Kendeou et al., 2019).

The proposed effectiveness of the myth-first format is also supported by time-based models of memory that emphasize the role of recency. Recent information is often found to be particularly strong in memory and easily retrieved (e.g., Baddeley & Hitch, 1993; Davelaar et al., 2005). Thus, a correction may have a stronger impact if placed after the misinformation. This phenomenon can be explained by models proposing that recall of recent information is facilitated by contextual overlap between encoding of recent information and its retrieval, driven by the temporal proximity of encoding and retrieval (e.g., Howard & Kahana, 2002; Sederberg et al., 2008). It can also be explained by models that assume that recently acquired representations are more temporally distinct due to lack of interference (Bjork & Whitten, 1974; Brown et al., 2007; Ecker, Brown, et al., 2015). In a misinformation context, Ecker, Lewandowsky, et al. (2015) presented people with multiple causes of an event, one of which was subsequently retracted. The authors found that the more recent cause tended to have the strongest influence on memory and reasoning and was more resistant to retraction than a cause presented earlier. Similarly, Vraga et al. (2020) presented participants with a series of Instagram posts that included a myth about climate change as well as a humorous factual correction of that myth, and manipulated the order of myth and fact. In the subsequent test, climate misperceptions were lower with a myth-first (where the fact was presented most recently) approach than a fact-first approach (where the myth was most recent). In sum, this account proposes that more recently acquired information is more impactful than information obtained earlier. This suggests that the most important information should be presented last, and in the case of debunking this is arguably the factual correction. Presenting factual information *after* the myth should thus promote optimal reliance on the factual information, rather than the false information.

### Correction prior to misinformation: the fact-first approach

Despite the popularity of the myth-first correction approach, a reverse-order fact-first approach—presenting the factual information prior to the misinformation—is often cited as preferable. It is argued that this approach emphasizes the fact rather than the myth and lets the factual information set the message framing (Cook & Lewandowsky, 2011; also see Lewandowsky et al., 2020). By presenting the fact first, subsequent information (including misinformation) should be understood and encoded primarily in the context of the factual information, rather than vice versa (Lakoff, 2010, see also Appelt et al., 2011; Weber et al., 2007). As the misconception is presented in contrast to the fact, it is argued that people should be more cognitively prepared and, therefore, more likely to encode the misinformation in a careful manner (Ecker et al., 2010; Kendeou & O’Brien, 2014).

There are also memory theorists who emphasize the importance of primacy, arguing that initially presented information is encoded into memory more strongly (e.g., Page & Norris, 1998; also see Farrell & Lewandowsky, 2002), receives more rehearsal (e.g., Tan & Ward, 2008), and benefits from temporal distinctiveness due to an absence of proactively interfering information (Brown et al., 2007; Ecker, Tay, et al., 2015). For example, in impression formation, more emphasis tends to be placed on early information received about a person, compared to information received later (e.g., Dreben et al., 1979; Sullivan, 2019). This account therefore suggests that the most important information should come first. Based on the presumptions underlying both primacy and framing effects, presenting factual information prior to the presentation of the misinformation should more effectively reduce misinformation beliefs compared to other corrective formats.

### The avoidance of familiarity effects: the fact-only approach

An even more extreme stance proposes not only deemphasizing the myth, but completely avoiding it. This is based on theoretical considerations that repeating the original misconception within the correction could impede its corrective impact due to the correction boosting the myth’s familiarity. This is thought to be problematic because people are more likely to believe information when it is familiar (the *illusory truth effect;* e.g., Begg et al., 1992; DiFonzo et al., 2016; Fazio et al., 2015). Some researchers have therefore argued that it may be beneficial to avoid myth repetition entirely to not increase myth familiarity, and therefore corrections should focus exclusively on the facts (e.g., Peter & Koch, 2016; also see Skurnik et al., 2005). Skurnik et al., (2007; as cited in Schwarz et al., 2007) presented participants with vaccine information aiming to reduce vaccine misconceptions. After a 30 minute delay, intent to vaccinate had increased for the facts-only format. By contrast, the “myths vs. facts” format backfired, resulting in *less* favorable vaccination attitudes compared to a control condition. The authors attributed this outcome to the corrections increasing myth familiarity. However, though the Skurnik et al. (2007) study is highly cited, it is difficult to evaluate given that it remains unpublished.

Initially, there were substantial concerns about such familiarity backfire effects (Cook & Lewandowsky, 2011; Lewandowsky et al., 2012). However, recent research has failed to produce the effect consistently (Ecker et al., 2017; Ecker et al., 2020; Ecker et al., 2011; Swire-Thompson et al., 2020; Swire-Thompson et al., 2021). Swire et al. (2017) investigated the effectiveness of myth corrections over the course of three weeks, in both young and older adults. While they found no evidence that correcting misinformation led to *backfire* effects relative to the pre-correction baseline, they concluded that familiarity could still be a contributing factor to the persistence of misinformation after a correction. This is because fact affirmations promoted more sustained belief change in comparison with myth retractions over the course of one week. Thus, framing a correction as a factual affirmation could be more effective than the myth-first or fact-first formats. For instance, rather than stating “the claim that people only use 10% of their brain is false,” one could focus just on the true statement that “people use all of their brain.” This method does not mention the original myth, therefore avoiding increased myth familiarity while still correcting the underlying misconception.

### Source confusion

An alternative explanation for the efficacy of Skurnik et al.’s (2007) facts-only format—other than reduced familiarity from avoiding repetition of the misconceptions—is that participants may have experienced less confusion at retrieval. Not only did participants have fewer items to remember (only three facts were affirmed compared to the three affirmed facts *and* three retracted myths in the myths vs. facts format), but the claims for which they received explanations were all true. It is possible that presenting all items with the same valence can help participants avoid a form of retrieval failure known as source confusion, where people confuse or misattribute the contextual details of a memory (Johnson et al., 1993; Schacter & Dodson, 2001). This is potentially an important phenomenon to consider when deciding how to present corrections: The common “myths vs facts” approach mixes true and false claims, which are then affirmed and refuted, respectively. However, one could choose to focus entirely on myth corrections (in either myth-first or fact-first format), or alternatively present only factual statements (using the fact-only format). In other words, presenting items as all myths or all facts may avoid potential source confusion and thus promote sustained belief change. This makes intuitive sense: Participants will be able to think back to the encoding phase knowing that all the claims encountered in that encoding context were either true or false.

### The current study

The current study aimed to assess whether the way a correction is configured influences its effectiveness and to tease apart the preceding theoretical alternatives. Across four experiments, participants were presented with corrections in a range of different conditions. To expand generalizability, materials varied substantially across experiments: Experiments 1 and 2 focused on corrections of misconceptions concerning climate change, whereas Experiments 3 and 4 extended this to misinformation regarding multiple topics including vaccines, alcohol, animals, the brain, and hypnotism. All experiments included both a myth-first and a fact-first correction condition, and Experiments 1, 3, and 4 included an additional fact-only condition. Experiments 3 and 4 also included fact affirmations to assess the potential impact of source confusion. While it was not possible to include a no-correction control condition in Experiments 1 and 2 for ethical reasons, such a control condition was included in Experiments 3 and 4.

Thus, this paper allows for a comprehensive evaluation of the relative effectiveness of different correction formats, which has implications for both application (e.g., design of debunking campaigns) and theorizing. If the myth-first format is better at reducing reliance on misinformation than the fact-first format, this would provide additional evidence that recency plays a significant role in the processing of corrections. By contrast, if the fact-first format is better at reducing reliance on misinformation, this would be additional evidence for the relevance of primacy and framing effects. If the fact-only condition is found to be most effective, this would highlight the importance of myth familiarity effects or that correction effectiveness may be negatively influenced by source confusion.

## Experiment 1

The aim of Experiment 1 was to investigate the efficacy of different correction formats, in order to determine whether one format is superior to others in reducing the continued influence effect. Participants were exposed to climate-related misinformation and then received a correction in either a myth-first, fact-first, or facts-only format in a one-way between-subjects design. An additional no-correction control group was not possible, because the correction discussed here was part of the experimental debrief of a separate study, which required the correction of real-world misinformation (Cook et al., 2017).^1^

### Method

#### Participants

A US representative sample (*N* = 588) was recruited through Qualtrics.com, selected by gender, age, and income demographics that we provided**.** There were 296 men and 292 women between 18 and 86 years of age, with a mean age of 47.63 years (*SD* = 14.55). Participants were randomly assigned to one of the three conditions.

#### Stimuli

##### Misinformation text

The misinformation text was an article about scientists debating the causes of climate change. The text first featured scientists who presented research supporting the claim that humans are causing global warming. This was followed by contrarian scientists rejecting human contributions to global warming and proposing alternative explanations. See Additional file 1: Section A for the full text.

##### Correction formats

Corrections were comprehensive explanations about the techniques used to cast doubt on climate science. Corrections targeted two specific myths, namely (a) that there is still substantial scientific debate regarding the cause of global warming and (b) that global warming is caused by the sun. These corrections existed in three formats. In the myth-first format, the myth was mentioned first (e.g., MYTH: There is no scientific consensus that humans are causing global warming) and the relevant fact was provided later (e.g., FACT: 97% of climate scientists agree humans are causing global warming). In the fact-first format, the order was reversed. Finally, in the fact-only format, participants only received the relevant facts. See Additional file 1: Section A for all correction texts.

##### Test phase

Eight items were used to measure participants’ climate perceptions and were presented in a fixed order. Four belief questions focused on the two myths directly and two questions focused on the associated facts. These questions used a five-point (1–5) Likert scale. Two inference questions asked participants to (a) estimate the percentage of climate scientists that agree human activity is causing global warming and (b) estimate the contribution from human CO2 emissions to increase temperature since 1880.

#### Procedure

All experiments were run using Qualtrics (Provo, Utah) surveys and were approved by the University of Western Australia’s Human Research Ethics Office. Participants initially received an ethics-approved information sheet and provided consent. Participants read the misinformation text, then answered a series of questions about climate change that formed part of a different study (Cook et al., 2017). Following this, participants received a correction as described above and responded to the belief and inference questions.

### Results

The myth items were reverse scored so that a composite could be created with the fact items. In other words, the six scale items (myths reverse-coded) were averaged to form a “climate perception score,” where higher endorsement equated to more accurate knowledge. The climate consensus and human contribution scores were analyzed separately. We conducted analyses using both null hypothesis significance testing and Bayes factors (BF). Bayes factors represent the relative evidence for one model over another. The findings can be expressed as either *BF*_10_ which quantifies support for the alternative hypothesis, or *BF*_01_ which quantifies support for the null hypothesis. A *BF* between 1 and 3 provides anecdotal evidence, 3 and 10 provides moderate evidence, 10 and 30 provides strong evidence, 30–100 provides very strong evidence, and a *BF* greater than 100 constitutes extreme evidence (Wagenmakers et al., 2018).

#### Climate perception score

Mean climate perception scores were *M* = 3.54 (*SD* = 0.76) for the myth-first, *M* = 3.64 (*SD* = 0.83) for the fact-first format, and *M* = 3.50 (*SD* = 0.83) for the fact-only format. A one-way ANOVA revealed a nonsignificant main effect of correction format, *p* = 0.186; *BF*_01_ = 10.44, indicating that the fact-first, facts-only, and myth-first formats were equivalent.^2^

#### Climate consensus

Mean climate consensus scores were *M* = 76.73 (*SD* = 31.03) for the myth-first format, *M* = 88.73 (*SD* = 21.11) for the fact-first format, and *M* = 88.06 (*SD* = 20.95) for the fact-only format.^3^ A one-way ANOVA revealed a significant main effect of correction format, *F*(2, 585) = 15.83; *p* < 0.001; *MSE* = 5.38; *η*_p_^2^ = 0.05; *BF*_10_ = 49,792.04, indicating that correction formats differed. Planned comparisons revealed myth-first format had a lower climate-consensus estimate than fact-first format, *F*(1, 391) = 24.12; *p* < 0.001; *MSE* = 5.94; η_p_^2^ = 0.06; *BF*_10_ = 9552.37, and the fact-only formats, *F*(1, 377) = 18.99; *p* < 0.001; *MSE* = 6.02; η_p_^2^ = 0.05; *BF*_10_ = 896.89. There was no significant difference between fact-first and fact-only conditions, *F* < 1.

#### Human contribution

The human contribution to climate change score was *M* = 57.70 (*SD* = 32.25) for the myth-first format, *M* = 63.96 (*SD* = 29.72) for the fact-first format, and *M* = 61.79 (*SD* = 31.12) for the fact-only format. There was no main effect of correction format, *p* = 0.132; *BF*_01_ = 7.55, suggesting that the different formats achieved comparable outcomes.

### Discussion

Experiment 1 tested the relative effectiveness of fact-first, myth-first, and fact-only formats in correcting climate misinformation. We found that the correction format did not differentially impact participants’ general climate myth perceptions or their perceptions of human contribution to climate change. Participants in the fact-first and fact-only conditions provided more accurate estimates of the expert consensus on anthropogenic climate change than participants in the myth-first condition. However, it is important to note that Experiment 1 measured expert consensus using a single item and therefore may have poor reliability (Swire-Thompson et al., 2021). Experiment 2 thus sought to replicate this finding in a real-world context using multi-item measures.

## Experiment 2

Experiment 2 was conducted as part of a massive open online course (MOOC), the inaugural edition of “Making sense of climate science denial” (https://www.edx.org/course/making-sense-of-climate-science-denial). In the MOOC, video lectures were designed around debunking climate myths and covered many aspects of climate change including fundamental climate science, psychological research into climate science denial, and effective techniques for responding to misinformation. We used a 2 × 2 within-subjects design with factors correction (pre-correction; post-correction) and format (myth-first; fact-first). A no-correction control group was not possible in this context as it was a requirement that all students would have access to all materials that were integral to the course. For four of the lectures in the course over weeks 2 to 5, two versions of each lecture were created (myth-first or fact-first). Students were assigned randomly to one of two groups, which received lectures 1 and 3 in the myth-first format and lectures 2 and 4 in the fact-first format, or vice versa. Thus, students received the same content, but the order of videos within a given lecture was manipulated, such that the myth or the fact was presented first. See Additional file 1: Section B for an illustration of the experimental design. Students completed an identical test survey in weeks 1 and 6 (pre-correction and post-correction) measuring their belief in the myths.

### Method

#### Participants

Surveys were open to all students enrolled in the MOOC but completion was voluntary. The total sample with complete records for both surveys was *N* = 1002.^4^ No demographic data were collected.

#### Stimuli

##### Correction formats

The corrections were embedded in a series of four online lectures that followed either the myth-first or fact-first format. This means that participants received the exact same content, but the order of videos within a given lecture was manipulated. The lectures specifically addressed four myths concerning contemporary temperature records, causal attribution of climate change to human actions, medieval temperatures, and species adaptation. For example, one video included the fact that observed patterns of climate change confirm human causation of recent global warming, as well as the myth that the sun is causing global warming. The myth-first format began with correcting the sun myth followed by the fact about human causation. By contrast, the fact-first format began with the fact about human causation then debunked the sun myth. The lectures can be found at https://www.skepticalscience.com/denial101x-videos-and-references.html.

##### Test phase

The test survey comprised a total of eight items, two questions per lecture. One targeted the relevant myth directly (e.g., “*Recent global warming has been caused by an unusually warm sun*”*—strongly disagree to strongly agree*) and one assessing the same belief but through a factual statement (e.g., “*Recent global warming has been caused mainly by human activity*”*—strongly disagree to strongly agree*), using five-point Likert scales. Given that students were randomly assigned to either receive lectures 1 and 3 in the myth-first format and lectures 2 and 4 in the fact-first format (or vice versa), this meant that there were four items from the myth-first lectures and four items from the fact-first lectures. See Additional file 1: Section B for all questions.

#### Procedure

Participants completed a pre-correction test survey to measure belief baseline in week 1 of the course. They then received the corrections in weeks 2–5 and completed the survey again (post-correction test) in week 6. The course was asynchronous, so the surveys were not conducted simultaneously; however, the course was timed, meaning that video content was published on a weekly basis and thus most participants viewed each week’s content in the same week.

### Results

The myth items were reverse-scored and pre-correction and post-correction scores were obtained for each condition (myth-first vs. fact-first). These scores were created by averaging the four items associated with each condition at each time point (henceforth: the knowledge scores).^5^ Mean knowledge scores across myth-first and fact-first formats and corrections are shown in Fig. 1. A 2 × 2 within-subjects ANOVA yielded a main effect of correction, *F*(1, 1001) = 318.12; *p* < 0.001; *MSE* = 0.20; η_p_^2^ = 0.24; *BF*_10_ = 4.23e + 67, indicating that climate knowledge increased from pre-correction to post-correction test. There was no main effect of format, *F* < 1; *BF*_01_ = 18.02, and no interaction, *F* < 1; *BF*_01_ = 37.18, indicating that myth-first and fact-first formats had no differential impact.

**Fig. 1:**
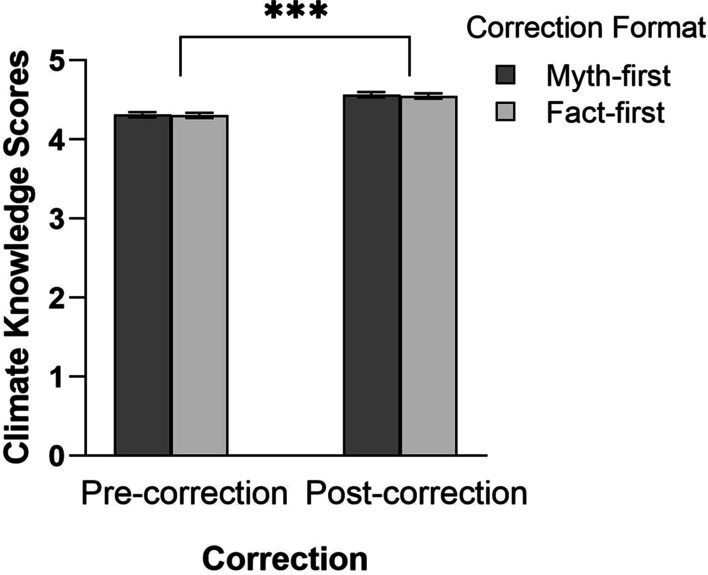
Average climate knowledge across formats and correction. Error bars denote 95% confidence intervals. *** *p* < .001

### Discussion

Experiment 2 tested fact-first and myth-first correction formats in four video lectures about climate misinformation. There was no main effect of the correction format on climate knowledge; when averaged over four lectures, both fact-first and myth-first lectures were equally effective. One observed limitation is that baseline composite knowledge scores were high. At the outset, participants scored 4.31/5, which rose to 4.56/5 post-correction. It is possible that if participants had more consistently believed in the misinformation (or disbelieved the factual information) pre-correction, one format may have been revealed to be superior. Experiment 3 was conducted to investigate the efficacy of diverse correction formats using (a) myths that were more likely to be believed, (b) a wider range of myths beyond climate change, (c) myth corrections in the context of independent fact affirmations in order to better approximate real-world fact-checking, and (d) a no-correction control condition.

## Experiment 3

The aim of Experiment 3 was to replicate the findings of Experiments 1 and 2 using a broader set of stimuli and using myths that were more likely to be initially believed to be true. Experiment 3 additionally extended the previous experiments by including facts that were topically related but independent of the presented myths (i.e., facts that were not simply the counterframe to the associated myth). Facts were always affirmed, while myths were always corrected. We use “explanation format” as an umbrella term for the format of both myth corrections and fact affirmations. Participants were presented with sets of myths and facts pertaining to various topics in an encoding phase, and different explanation formats were used for each set. Participants were then asked to rate their beliefs in the presented claims in a test phase.

We used five different explanation formats. First, the *standard format* replicated the standard “myths vs. facts” pamphlet, where both myth and fact claims regarding a particular topic were first presented, and each was followed by a false/true label and an explanation (i.e., a correction or affirmation). In other words, for the myths, this is the myth-first condition. Second, a *reverse* format placed the explanation as to why the myth/fact is false/true prior to the false/true tag and claim itself. For myths, this was the fact-first condition. Third, in the *facts-only* format, all myths were re-framed as factual statements, thus avoiding myth repetition. Fourth, a *myths-only* format corrected myths in an identical fashion to the standard (myth-first) condition, but the filler facts were omitted to avoid potential source confusion. Finally, we included a no-explanation *control* condition, which involved no encoding phase and only belief ratings at test. For an illustration of the components included in each condition, see Table 1.

**Table 1:** Number of myths/facts and component order in each explanation condition

Format	Items presented	Order of components
Standard	3 myths 3 facts	(1) claim (2) false/true label (3) retraction/affirmation
Reverse order	3 myths 3 facts	(1) retraction/affirmation (2) false/true label (3) claim
Facts-only	3 myths (framed as facts) 3 facts	(1) claim (2) false/true label (3) retraction/affirmation
Myths-only	3 myths 0 facts	(1) claim (2) false label (3) retraction
No explanation control	0 myths 0 facts	NA

### Method

Experiment 3 used a 2 × 5 within-subjects design, with factors item type (myth vs. fact) and explanation format (standard vs. reverse vs. facts-only vs. myths-only vs. control). Assignment of claim sets to explanation formats was counterbalanced. We were primarily interested in the efficacy of myth corrections but also present the data from fact affirmations.

#### Participants

Participants were 99 Amazon Mechanical Turk workers, who were paid $3 for a 25-min survey. To qualify, workers had to have completed a minimum of 1,000 so-called “human intelligence tasks” on the platform. There were 38 women and 61 men between 21 and 68 years of age, with a mean age of 34.26 (*SD* = 10.13).

#### Stimuli

There were five sets of items, each consisting of three myths and three facts. Each set was concerned with a different topic: the brain, alcohol, animals, hypnotism, and the flu. Stimuli from the flu topic were taken directly from Schwarz et al. (2007). An example myth in standard, reverse-order, and facts-only formats can be found in Table 2. Belief was rated on an 11-point (0–10) scale ranging from “Definitely True” to “Definitely False.” For every item, there was also an inference question designed to be a less direct measure of belief. These were included because people can rely on misinformation in their inferential reasoning even when they exhibit successful discounting in direct belief ratings (see Ecker et al., 2011). The inference questions were also rated on an 11-point scale, with the specific scale-value range varying from item to item (i.e., some were on a 0–10 scale, others were on a 0–20% scale with 2% increments, etc.). The full list of stimuli is provided in Additional file 1: Table S1 in Section C. Compared to the corrections in Experiments 1 and 2, the corrections in Experiment 3 were more concise. Where corrections in Experiments 1–2 were approximately 560 words, the current corrections were approximately 65 words.

**Table 2:** Example of a correction in standard format, reverse-order format, and facts-only frame, as well as an example inference question and belief rating

Correction format	Example
Standard correction	Alcohol promotes sleep
	Alcohol promotes sleep—MYTH
	Alcohol disturbs sleep: Drinking alcohol before bed leads to REM sleep being disrupted. This is followed by abnormally shallow sleep, causing multiple awakenings. The more alcohol consumed prior to sleep, the more pronounced these effects are. So, although alcohol may help the onset of sleep, sleep quality is adversely affected
Reverse-order correction	Alcohol disturbs sleep: Drinking alcohol before bed leads to REM sleep being disrupted. This is followed by abnormally shallow sleep, causing multiple awakenings. The more alcohol consumed prior to sleep, the more pronounced these effects are. So, although alcohol may help the onset of sleep, sleep quality is adversely affected
	It is a MYTH that alcohol promotes sleep
Facts-only frame	Sleep is adversely affected by alcohol
	Sleep is adversely affected by alcohol—FACT
	Alcohol disturbs sleep: Drinking alcohol before bed leads to REM sleep being disrupted. This is followed by abnormally shallow sleep, causing multiple awakenings. The more alcohol consumed prior to sleep, the more pronounced these effects are. So, although alcohol may help the onset of sleep, sleep quality is adversely affected
Inference question	If your insomniac friend told you they were planning on drinking two glasses of wine before bed to help them sleep, would you advise them otherwise? (0, *Definitely not* – 10, *Definitely*)
Belief rating	How much do you believe this claim:
	Alcohol promotes sleep (0, *Not at all –* 10*, Very much so)*

*Note*: The myth-only condition was identical to the standard correction, except that the three myths were presented on their own, without any of the three facts

#### Procedure

In the encoding phase participants were presented with four of the five sets of items—the non-presented set was allocated to the control condition. In other words, if the sets regarding the brain, alcohol, animals, and the flu were corrected/affirmed (each using different formats), then the remaining set regarding hypnotism would not be presented at all and would act as a control. Assignment of claim sets to explanation formats was counterbalanced and presented in a random order; items within each set were also presented in random order. All items in the experimental sets were retracted/affirmed using one of the four explanation formats. The test phase followed immediately after the encoding phase. The test involved a block of inference questions (one per item, in random order) and a block of direct belief ratings.

### Results

#### Belief ratings

In contrast to Experiments 1 and 2 that combined the reverse-scored myths and facts into one climate perception score or climate knowledge score, in Experiment 3 we created a myth composite score from the myth items and a fact composite score from the fact items. As can be seen in Fig. 2, all explanation formats led to belief change, and the efficacy of corrections and affirmations was largely independent of the format used. In other words, the alternative explanation formats (i.e., reverse order, facts-only, and myths-only) were equivalent to the standard format. For fact items, the myths-only condition was not expected to differ from control because it did not provide any fact affirmations.

**Fig. 2:**
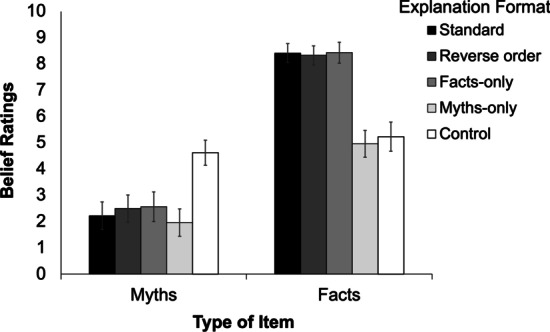
Belief ratings across conditions in Experiment 3. Error bars denote 95% confidence intervals

A within-subjects ANOVA on myth belief ratings yielded a main effect of explanation format, *F*(2.98, 291.64) = 34.40; *p* < 0.001; *MSE* = 4.34; η_p_^2^ = 0.26; *BF*_10_ = 3.24e + 21, showing that ratings differed across explanation formats. Table 3 shows Holm–Bonferroni corrected comparisons, which confirmed that (a) all formats differed from control, and (b) the standard format did not differ from the other formats. Bayes factors provided very strong evidence that all correction conditions differed from control, and anecdotal evidence that there was no difference between the standard format and the reverse-order, facts-only, or myths-only conditions.

**Table 3:** Planned comparisons on myth belief ratings in Experiment 3

	Standard	Reverse order	Facts-only	Myths-only
Reverse order	*F* = 2.03 *p* = .16 *BF*_01_ = 2.48			
Facts-only	*F* = 2.31 *p* = .13 *BF*_01_ = 2.26			
Myths-only	*F* = 2.09 *p* = .15 *BF*_01_ = 2.50			
Control	*F* = 62.00 *p* < .001* *BF*_10_ = 8.44e + 9	*F* = 57.87 *p* < .001* *BF*_10_ = 7.54e + 8	*F* = 35.78 *p* < .001* *BF*_10_ = 1.72e + 6	*F* = 73.56 *p* < .001* *BF*_10_ = 5.81e + 11

All df1 = 1, df2 = 98; * indicates significance after Holm–Bonferroni correction

There was also a main effect of explanation format on fact belief ratings, *F*(2.72, 266.15) = 68.91; *p* < 0.001; *MSE* = 6.90; η_p_^2^ = 0.41; *BF*_10_ = 7.09e + 43. See Additional file 1: Table S2 for Holm–Bonferroni corrected comparisons of fact ratings, which showed that all formats differed from control (except, as expected, the myth-only condition), and the standard format did not differ from the other formats.

#### Inference scores

As can be seen from Fig. 3, the inference scores closely mirrored the belief ratings. We conducted a within-subjects ANOVA on the myth inference scores. The main effect of explanation format, *F*(3.77, 369.06) = 11.69; *p* < 0.001; *MSE* = 4.03; η_p_^2^ = 0.11; *BF*_10_ = 1.87e + 6, indicated that belief ratings differed across explanation formats. Similarly, a within-subjects ANOVA on the fact inference scores revealed a significant main effect of explanation format, *F*(3.42, 335.16) = 67.79; *p* < 0.001; *MSE* = 4.05; η_p_^2^ = 0.41; *BF*_10_ = 5.55e + 42.

**Fig. 3:**
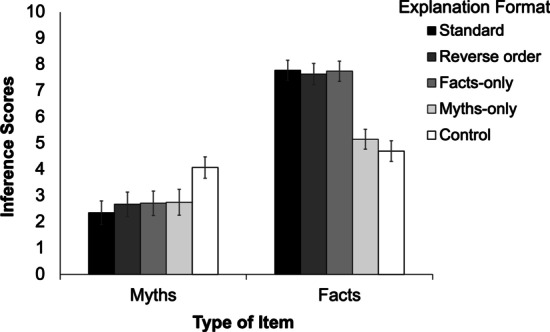
Inference scores across conditions in Experiment 3. Error bars denote 95% confidence intervals

Tables 4 and Additional file 1: Table S3 show planned comparisons for myths and facts, respectively. Paralleling the belief ratings, all corrections and affirmations were effective relative to control, and there were no significant differences between the alternative explanation formats (i.e., reverse, facts-only, and myths-only) and the standard format, again with the to-be-expected exception of the myths-only condition for fact items. Given the similarity between Experiments 3 and 4, discussion of Experiment 3 results will be deferred until the Experiment 4 data are presented. Experiment 4 sought to replicate Experiment 3 and use longer retention intervals between encoding and test phase.

**Table 4:** Planned comparisons on myth inference scores in Experiment 3

	Standard	Reverse order	Facts-only	Myths-only
Reverse order	*F* = 1.60 *p* = .21 *BF*_01_ = 2.69			
Facts-only	*F* = 2.05 *p* = .16 *BF*_01_ = 2.49			
Myths-only	*F* = 2.70 *p* = .10 *BF*_01_ = 1.87			
Control	*F* = 37.77 *p* < .001* *BF*_10_ = 4.07e + 6	*F* = 21.61 *p* < .001* *BF*_10_ = 7051	*F* = 21.73 *p* < .001* *BF*_10_ = 5333	*F* = 15.44 *p* < .001* *BF*_10_ = 821.47

All df1 = 1, df2 = 98; * indicates significance after Holm–Bonferroni correction

## Experiment 4

Experiment 4 extended Experiment 3 by using longer retention intervals (i.e., one-week and three-week study-test delay), to explore whether belief change is independent of explanation format over a longer term.

### Method

Experiment 4 used a 2 × 2 × 5 between-within design, with the within-subjects factors item type and explanation format, and the between-subjects factor retention interval (one week vs. three weeks).

#### Participants

Participants were *N* = 198 undergraduate students from the University of Western Australia, who received course credit for participation. There was only one participant who did not complete the study. Our final sample (*N* = 197) included 130 women and 67 men between 15 and 59 years of age, with a mean age of 20.37 (*SD* = 5.67).

#### Stimuli and procedure

Stimuli were identical to Experiment 3; the procedure was similar, although the encoding phase took place in laboratory testing booths. Participants were not part of another study and left after the encoding phase had been completed. The test phase followed either one or three weeks after the encoding phase and was emailed to participants to complete. It was administered in an online format as in Experiment 3 to keep participation rates high.

### Results

#### Belief ratings

As shown in Fig. 4, all explanation formats were effective at reducing belief in comparison with the control condition, even after a three-week period.^6^ We first conducted a 2 × 5 within-between ANOVA with factors retention interval (one week vs. three weeks) and explanation format (standard vs. reverse vs. facts-only vs. myths-only vs. control) on the myth belief ratings, revealing two main effects.

**Fig. 4:**
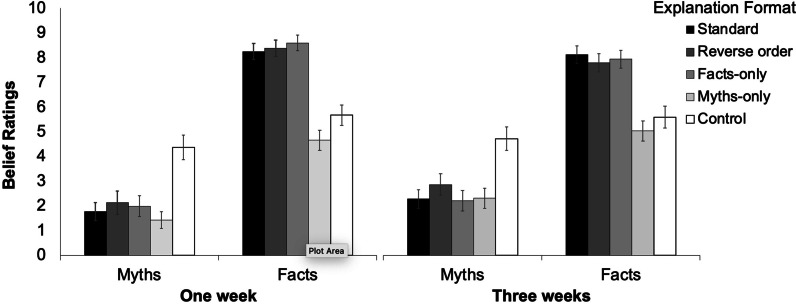
Belief ratings across conditions in Experiment 4. Error bars denote 95% confidence intervals

The main effect of retention interval, *F*(1,195) = 8.25; *p* = 0.005; *MSE* = 8.71; η_p_^2^ = 0.04; *BF*_10_ = 6.10, indicated that myth belief increased between one and three weeks. The main effect of explanation format, *F*(4,780) = 71.68; *p* < 0.001; *MSE* = 3.37; η_p_^2^ = 0.27; *BF*_10_ = 6.53e + 48, indicated that belief ratings differed across explanation formats. The myth planned comparisons are presented in Table 5. To limit the number of comparisons, we collapsed over retention-interval conditions, given there was no retention interval × explanation format interaction. For myths, the results confirmed that all correction formats differed from control and that the standard format had greater efficacy compared to the reverse-order format, but not the facts-only and myths-only formats. According to the Bayes factor analyses, there was moderate evidence that the standard format was more effective than the reverse-order format, and moderate evidence that the standard format did not differ from the facts-only or myth-only formats.

**Table 5:** Planned comparisons on myth belief ratings in Experiment 4

	Standard	Reverse order	Facts-only	Myths-only
Reverse order	*F* = 7.36 *p* = .007* *BF*_10_ = 3.62			
Facts-only	*F* = .24 *p* = .625 *BF*_01_ = 8.01			
Myths-only	*F* = 1.11 *p* = .293 *BF*_01_ = 5.05			
Control	*F* = 154.99 *p* < .001* *BF*_10_ = 1.02e + 27	*F* = 93.11 *p* < .001* *BF*_10_ = 2.57e + 16	*F* = 125.76 *p* < .001* *BF*_10_ = 1.05e + 23	*F* = 185.67 *p* < .001* *BF*_10_ = 2.12e + 30

All df1 = 1, df2 = 195; * indicates significance after Holm–Bonferroni correction

For the facts we similarly conducted a 2 × 5 within-between ANOVA with factors retention interval (one week vs. three weeks) and explanation format (standard vs. reverse vs. facts-only vs. myths-only vs. control). We found one main effect of explanation format, *F*(4,780) = 174.75; *p* < 0.001; *MSE* = 3.02; η_p_^2^ = 0.47, indicated that belief differed across conditions. As can be seen from Additional file 1 Table S4, all affirmation formats differed from control. The standard format did not differ from the other affirmation formats, with the exception of the myths-only condition, which did not feature fact affirmations and indeed produced *lower* belief ratings than control. We also found an interaction of retention interval and explanation, *F*(4,780) = 8.76; *p* = 0.023; *MSE* = 3.02; η_p_^2^ = 0.02, with planned comparisons revealing that the standard format promoted sustained belief change *more* than the reverse order or fact-only conditions, *F*(1,195) = 4.32; *p* = 0.039.

#### Inference scores

Mean inference scores are provided in Fig. 5. First, a 2 × 5 between-within ANOVA with factors retention interval and explanation format was performed on participants’ mean myth inference scores. There was no main effect of retention interval, *p* = 0.085; *BF*_01_ = 1.97, indicating that scores remained relatively stable over time. There was a main effect of explanation format, *F*(4, 780) = 33.98; *p* < 0.001; *MSE* = 2.92; η_p_^2^ = 0.15; *BF*_10_ = 2.13e + 23, indicating that conditions differed.

**Fig. 5:**
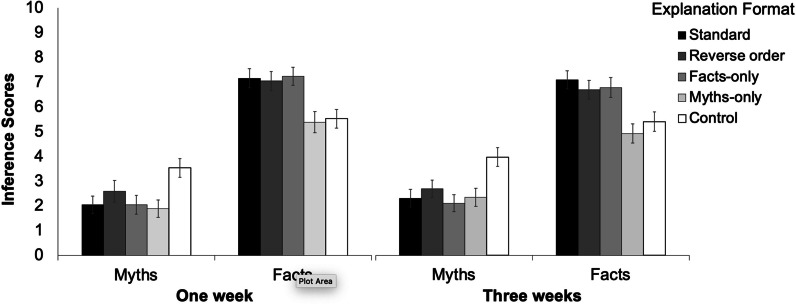
Inference scores across conditions in Experiment 4. Error bars denote 95% confidence intervals

Analogous to the belief ratings, planned comparisons were performed on myth inference scores (see Table 6). All correction formats had significantly lower inference scores than control. The standard format had lower inference scores than the reverse-order format, but not the facts-only and myths-only correction formats.

**Table 6:** Planned comparisons on myth inference scores in Experiment 4

	Standard	Reverse order	Facts-only	Myths-only
Reverse order	*F* = 7.15 *p* = .008* *BF*_10_ = 4.20			
Facts-only	*F* = .40 *p* = .528 *BF*_01_ = 7.73			
Myths-only	*F* = .14 *p* = .712 *BF*_01_ = 8.63			
Control	*F* = 94.23 *p* < .001* *BF*_10_ = 2.52e + 16	*F* = 34.43 *p* < .001* *BF*_10_ = 2.67e + 6	*F* = 96.50 *p* < .001* *BF*_10_ = 1.38e + 17	*F* = 91.48 *p* < .001* *BF*_10_ = 4.08e + 16

All df1 = 1, df2 = 195; * indicates significance after Holm–Bonferroni correction

Next, a 2 × 5 within-between ANOVA was performed on fact inference scores. There was a main effect of retention interval, *F*(1,195) = 5.14; *p* = 0.025; *MSE* = 4.04; η_p_^2^ = 0.03; *BF*_10_ = 0.52, indicating that scores slightly decreased between one and three weeks, and a main effect of explanation format, *F*(4,780) = 36.9; *p* < 0.001; *MSE* = 3.69; η_p_^2^ = 0.19; *BF*_10_ = 1.93e + 34, indicating that affirmation conditions were associated with greater inference scores. Planned comparisons are shown in Additional file 1: Table S5. All conditions differed from control, apart from the myths-only format (which featured no factual affirmations). The standard format did not differ from the other affirmation formats, with the exception of the myths-only condition.

### Discussion

Experiments 3 and 4 investigated the relative efficacy of various explanation formats. Experiment 3 indicated that immediately after corrections belief change was independent of the specific format of explanation used. Experiment 4 largely replicated these results, with the exception that the standard myth-first format resulted in stronger myth-belief reduction compared to the reverse-order (fact-first) format after a delay. This suggests that the standard format of leading with and then correcting a myth may be preferable to a correction that leads with the factual alternative. One limitation of Experiment 4 is that we did not explicitly ask the participants to refrain from looking up additional information online. However, if participants had looked up the items, they would likely find corroborating evidence that the misinformation was indeed false, or facts indeed true. Furthermore, given that the design was within-subjects, this would not have impacted conditions differentially.

Experiment 4 also highlighted a potential downside of focusing communications on myths only: The myths-only condition resulted in *lower* fact belief than control. In other words, when participants were not presented with any fact affirmations, they were more likely to assume that any information regarding the topic was false. In contrast to the *implied truth effect,* where flagging a subset of claims as false can increase belief in other false claims that are not flagged (Pennycook et al., 2020), our observed effect is likely driven by the close thematic relation between claims. The finding may therefore be an artifact of the experimental test situation, as participants in this condition were asked to concurrently rate their beliefs in claims that were refuted and related claims not previously presented. It is uncertain whether our effect may be a concern for real-world debunking, given that people are not often presented with a closed set of thematically related items and required to draw conclusions about other claims.

## General discussion

Across four experiments, the current study aimed to assess the relative efficacy of correction configurations with a particular focus on reducing myth beliefs. The results indicated that all corrections substantially decreased belief in misinformation in comparison with control conditions. This provides further evidence that the familiarity backfire effect should not be considered a concern when correcting misinformation (aligning with Ecker et al., 2020; Ecker et al., 2011; Swire et al., 2017; Swire-Thompson et al., 2021). The impact of a correction on beliefs and inferential reasoning scores was largely independent of the specific format used, and there was no single format that was clearly more effective than another. In general, it appears that as long as the key ingredients of an effective correction were presented, order did not make a considerable difference. To illustrate, the largest effect size elicited when comparing correction formats in Experiment 4 was η_p_^2^ = 0.02, yet when the control condition was included the observed effect size was 10 times greater (η_p_^2^ = 0.28). This highlights that simply providing corrective information, regardless of format, is far more important than how the correction is presented.

When focusing on the observed differences between the correction formats, the clearest evidence for any potential relative superiority emerged in Experiment 4, which found that with a delayed retention interval, the standard myth-first format was more effective at myth correction than the fact-first format. This aligns with the literature on refutational texts (e.g., Guzzetti et al., 1993), the notion that co-activation and conflict detection are conducive to knowledge revision (Ecker et al., 2017; Kendeou et al., 2019), and time-based models of memory that emphasize the role of recency (e.g., Baddeley & Hitch, 1993; Brown et al., 2007). Future research should replicate this finding and tease apart which is the more relevant underlying mechanism: co-activation or recency.

By contrast, Experiment 1 found that the fact-first format was more effective at instilling accurate knowledge regarding the expert consensus on climate change. However, given that this finding emerged in only one measure, it provides weak evidence for the importance of primacy (Farrell & Lewandowsky, 2002) and the notion that by presenting the fact-first, the subsequent misinformation can be understood in the context of the factual information frame (Lakoff, 2010). We therefore argue that the evidence overall does not support a significant role for primacy or fact-first framing in the processing of corrections. Likewise, the notion that myth familiarity (Skurnik et al., 2007) or source confusion (Schacter & Dodson, 2001) are key factors in the correction of misinformation was also not supported, given that the myth-first approach was found to be as effective or even more effective than other formats, and the fact-only approach did not lead to superior belief updating relative to other formats (aligning with Winters et al., 2021).

A secondary finding from Experiment 4 is that participants who were only presented with corrected myths (and no affirmed facts) subsequently rated the facts as less true than the control condition. In contrast to the *implied truth effect,* where flagging a subset of claims as false can increase belief in unflagged false claims (Pennycook et al., 2020), our observed effect is likely driven by the close thematic relation between claims. For instance, if all items regarding vaccines are presented as false, participants might have reasonably assumed that any new items regarding vaccines were also false. It is an open question whether this is a real-world concern and should be tested in the context of myth-versus-fact health pamphlets and other thematically related closed information sets. While presenting “balanced” arguments may not always be appropriate and can at times be misleading (e.g., false-balance media coverage; see Cook et al., 2017; Dixon & Clarke, 2013), truthfully explaining both facts and fiction in an educational setting might well give people a more nuanced view of a subject.

Future research should directly investigate whether different types of misconceptions benefit from different correction formats. For instance, it has been suggested that the fact-first approach may be more effective if there is a pithy replacement fact available that is novel or “sticky” (Heath & Heath, 2007; Lewandowsky et al., 2020). The replacement fact in Experiment 1—that there is an expert consensus on climate change—may represent such a “sticky” fact, given that public perception of the expert consensus remains low (Leiserowitz et al., 2020). Future investigations will require development of a more sophisticated conceptualization of “stickiness” in order to pinpoint the underlying mechanism—whether it be that the information is more salient, clear, memorable, or that it elicits greater attention or surprise in the individual.

Another consideration is whether demand characteristics are responsible for the reduction in misinformation belief post-correction, given that attempts to be a “good subject” could lead participants to report belief change without actually altering their beliefs. We do not think that this possibility is a primary driver of the observed effects for several reasons. First, Ecker et al. (2010) found that variance in continued influence effects was not due to a desire to please the experimenter. Second, various studies have found that effects of corrections are comparable whether direct belief measures are used or more indirect inference measures, which are arguably less prone to demand characteristics (e.g., Ecker et al., 2020). Finally, if we expect demand characteristics to be driving changes in expressed belief, participants’ memory for a veracity label and their belief in a claim should be identical, yet studies have shown that these often dissociate (e.g., O'Rear & Radvansky, 2020). However, future research should further examine the interplay of memory and belief and the role of demand characteristics when investigating the correction of misinformation.

Despite the absence of an “optimal” correction method, several practical fact checking recommendations can be made based on the results of the current study. For instance, this study provides further evidence that repeating the misconception within the retraction is not problematic, consistent with previous recommendations (Swire et al., 2017; Ecker et al., 2017; contrary to Skurnik et al., 2005). In other words, it is acceptable and could even be beneficial to repeat the myth explicitly when debunking it. However, while the order of elements is unlikely to be largely consequential, it is still important that the misconception is not described alone without the correction being saliently paired, (for example in new headlines; Fazio et al., 2015).

In sum, our findings suggest that all corrections—regardless of format—are effective at fostering belief change and that no correction format elicits backfire effects. These experiments should be replicated and extended prior to issuing firm policy recommendations, and the current paper provides a theoretical framework that might provide a useful scaffold for future research. However, we present initial evidence for fact-checkers that the format of their correction is not crucial to effective belief updating. It may therefore be more important to focus on getting corrections (of any format) to the people most likely to hold relevant false beliefs, especially where such misconceptions have the greatest potential for harm.

## Supplementary Information

**Additional file 1:** Supplementary materials including stimuli and additional analyses.

## Data Availability

Data will be publicly available on the website Dryad.com upon publication.

## Footnotes

1 The correction discussed here was part of the experimental debrief of a separate study reported in Cook et al. (2017). The study had four groups of participants; some received additional information beforehand on the scientific consensus on climate change and/or the fake-debate strategy used by the tobacco industry to confuse the public about the level of consensus; an additional control group received no misinformation. For present purposes, the grouping factor is irrelevant and including it as an additional factor in our analyses did not change the outcome.

2 Here and in all following analyses, degrees of freedom were corrected using Huynh–Feldt estimates of sphericity, where appropriate.

3 Reflection and square root transformation were applied to the data due to negative skew. This resulted in acceptable levels of skew and kurtosis. Levene’s test of homogeneity of variances indicated that the assumption was not satisfied (*p* < .05); however, if sample sizes across conditions are approximately equal, ANOVA is fairly robust to such violations (Boneau, 1960; Glass, Peckham, & Sanders, 1972).

4 This excludes participants (*n* = 66) who used an additional “Prefer not to answer” response option in any question; this option was included for ethical reasons only. The pre-correction sample with complete data for the eight climate items was *N* = 5291; descriptive statistics for the full pre-correction sample can be found in Additional file 1: section B.

5 We acknowledge that the grouping of lecture topics introduces a slight confound, as lectures 1 and 3, as well as lectures 2 and 4, always had the same format; practicality constraints prevented a more complex, unconfounded design.

6 Due to an error with randomization, the “alcohol” topic was not presented to participants in the standard format. Rather than excluding those participants’ data entirely, missing data were imputed, using the mean of the available scores from the standard-format condition, and taking into account score differences between alcohol and non-alcohol claims, as well as individual response tendencies to corrected myths and affirmed facts from the remaining conditions. For details, as well as results from analyses excluding these participants (which yielded equivalent results), see Additional File 1: section C.
