# Supplementary material for: Correction format has a limited role when debunking misinformation
Source: Cogn Res Princ Implic. 2021 Dec 29;6:83. doi: 10.1186/s41235-021-00346-6 (PMC8715407; doi:10.1186/s41235-021-00346-6)
Supplement: Supplementary file 1 — Additional file 1: Supplementary materials including stimuli and additional analyses. [file 41235_2021_346_MOESM1_ESM.pdf]

Supplemental Material
Evidence for a Limited Role of Correction Format when Debunking Misinformation

Supplement A

Experiment 1 Texts

Control text

Why there is a 'human' in 'humanities'.

The term humanities refers to the academic disciplines that study human culture and the human condition. The humanities engage in the intellectual activity of studying the meaning-making practices of human culture, past and present. Humanities scholars argue that the human experience cannot be adequately captured by facts and figures alone. Humanities research therefore employs methods that are comparative, interpretive, critical-analytical, or speculative in nature. The humanities often have a historical element, and aim to learn from the past to understand the present and prepare for the future. The subjects relate to manifestations of what makes us human, such as language, literature, history, art, philosophy, and law. The humanities are mainly interested in raising questions, rather than providing answers. They do not search for the ultimate correct answer to a question, but instead explore the issues and details that surround it. Context is always important, and context can be social, historical, political, cultural or ethnic.

Misinformation text

Scientists debate causes of climate change.

Scientists have released a controversial new report concluding that humans are causing most of global warming. The report is an assessment of different lines of evidence, including thermometer measurements, satellite data and observations of ocean temperature. "There are a number of distinctive patterns being observed in our climate," said climate scientist Dr Jason Griffiths from the University of California, one of the lead authors of the report. "Put together, these patterns strengthen the evidence that humans are causing global warming. They also eliminate other possible natural causes." The report lists different lines of evidence supporting human-caused global warming. "Satellites are measuring less heat escaping to space because heat trapping greenhouse gases are absorbing more heat," said Dr Jones. "Other expected patterns of greenhouse warming have also been observed. For example, winters warming faster than summers is a characteristic pattern of warming from greenhouse gases." However, other scientists have criticised the report for not including the views of scientists who disagree with the mainstream position. Dr Bertram Pearse, a climate expert from George Mason University, commented, "This report ignores the fact that eminent climate scientists, who have published hundreds of peer-reviewed papers between them, argue that humans have not had a significant effect on climate. Climate change is still hotly debated among scientists." Dr Smith has published studies questioning the link between human activity and climate change. "A key driver of climate is variations in solar activity," Smith explains. "The sun provides almost all the energy in our climate system. When the sun

gets warmer, our planet gets warmer. Over the last few decades, the sun has been unusually warm, achieving its warmest levels in 1,150 years."

Consensus text

The Scientific Consensus on Global Warming

Climate research shows that the temperature of the Earth has been increasing every decade since the 1970s. It is also established that the burning of fossil fuels releases heat-trapping greenhouse gases into the atmosphere. Greenhouse gas emissions have been directly linked to global warming and climate change. A recent survey of scientists has found that the greater their expertise in climate science, the stronger their agreement on human-caused global warming. Among climate scientists who are actively publishing research into climate, 97 out of 100 agree that global warming is a direct consequence of the burning of fossil fuels by humans. Overwhelming agreement is also found in published climate research. An analysis of 21 years of climate research found that 97.1% of relevant papers agree with the consensus. Papers rejecting the consensus are a vanishingly small minority. Surveys of the climate science community and analysis of published climate research both find the same result. Based on the evidence, 97% of climate scientists have concluded that human-caused climate change is happening. Fossil fuel and political groups try to cast doubt on climate science, in order to delay regulation of the fossil fuel industry. They do this by manufacturing the appearance of an ongoing scientific debate. The "fake debate" strategy was pioneered by the tobacco industry in the 1970s. They used doctors and scientists to reassure the public that smoking didn't cause health problems. These spokespeople were either non-experts or among the small minority of scientists who dissented from the scientific consensus that smoking is bad for you. However, in the public's eye, this conveyed the appearance of ongoing scientific debate. The "Tobacco Strategy" is being used again, but this time to cast doubt on climate science. Less than 3% of climate scientists disagree with the consensus position that humans are causing global warming. However, when the media present the views of a dissenting scientist alongside a mainstream scientist, the public comes away with the mistaken impression of a 50:50 debate. Ironically, the journalistic standard of giving both sides equal weight has ended up distorting the state of climate science.

Inoculation text

The Scientific Consensus on Global Warming

Fossil fuel and political groups try to cast doubt on climate science, in order to delay regulation of the fossil fuel industry. They do this by manufacturing the appearance of an ongoing scientific debate. The "fake debate" strategy was pioneered by the tobacco industry in the 1970s. They used doctors and scientists to reassure the public that smoking didn't cause health problems. These spokespeople were either non-experts or among the small minority of scientists who dissented from the scientific consensus that smoking is bad for you. However, in the public's eye, this conveyed the appearance of ongoing scientific debate. The "Tobacco Strategy" is being used again, but this time to cast doubt on climate science. Less than 3% of climate scientists disagree with the consensus position that humans are causing global warming. However, when the media present the views of a dissenting scientist alongside a mainstream scientist, the public comes away with the mistaken impression of a

50:50 debate. Ironically, the journalistic standard of giving both sides equal weight has ended up distorting the state of climate science.

Experiment 1 Correction Formats

Fact then myth

Thank you for taking part in today's study. The session is almost complete but before you finish, it is important that you read the following debriefing information very carefully—you will be assessed on its contents shortly. Please note that you were exposed to misinformation in this survey. Some of the information in the article "Scientists debate causes of climate change" was in fact misleading and misrepresents the facts. The aim of the current study is to establish whether it is possible to protect members of the general public from deliberately misleading and inaccurate information—known as "misinformation". The following content explains how the previous article misrepresented the facts. Please read the content carefully, as you will be asked questions about it afterwards.
FACT: 97% of climate scientists agree humans are causing global warming.
Several surveys of climate scientists have found that 97% agree that humans are causing global warming. Also, over the last 21 years, 97% of scientific papers that state a position on whether humans were causing global warming agree with the consensus. There is overwhelming scientific agreement that humans are driving recent global warming.
Some groups try to cast doubt on human-caused global warming. They do this by manufacturing the appearance of an ongoing scientific debate. The "fake debate" strategy was pioneered by the tobacco industry in the 1970s. They used doctors and scientists to reassure the public that smoking didn't cause health problems. These spokespeople were either non-experts or among the small minority of scientists who dissented from the scientific consensus that smoking is bad for you. However, in the public's eye, this conveyed the appearance of ongoing scientific debate. The "Tobacco Strategy" is being used again, but this time to cast doubt on climate science. Less than 3% of climate scientists disagree with the consensus position that humans are causing global warming. However, when the media present the views of a dissenting scientist alongside a mainstream scientist, the public comes away with the mistaken impression of a 50:50 debate. Ironically, the journalistic standard of giving both sides equal weight has ended up distorting the state of climate science.
FACT: Climate patterns confirm human-caused global warming, rule out the sun.
A number of climate patterns confirm that heat-trapping greenhouse gases are causing global warming. Winters are warming faster than summers and nights are warming faster than days. The upper atmosphere is cooling while the lower atmosphere warms. These patterns rule out the sun as a potential cause of global warming. They also constitute a 'fingerprint' for the fact that humans are causing global warming through greenhouse gas emissions. Despite the evidence, a persistent myth is that the Sun is causing global warming. People persist in this myth by cherry picking data. For example, they look at times in the Earth's past when temperature and solar activity moved in the same direction. But they ignore recent data. In the last few decades of global warming, solar activity and climate have moved in opposite directions. Surface temperatures have increased and global temperatures hit the hottest on record in 2010. At the same time, the Sun has shown a slight cooling trend. In 2009, solar

activity reached its lowest levels in over a century. If anything, the drop in solar activity has had a slight cooling influence on climate in recent decades.

Myth then fact

Thank you for taking part in today's study. The session is almost complete but before you finish, it is important that you read the following debriefing information very carefully—you will be assessed on its contents shortly. Please note that you were exposed to misinformation in this survey. Some of the information in the article "Scientists debate causes of climate change" was in fact misleading and misrepresents the facts. The aim of the current study is to establish whether it is possible to protect members of the general public from deliberately misleading and inaccurate information—known as "misinformation". The following content explains how the previous article misrepresented the facts. Please read the content carefully, as you will be asked questions about it afterwards.

MYTH: There is no scientific consensus that humans are causing global warming.

FACT: Some groups try to cast doubt on human-caused global warming.

They do this by manufacturing the appearance of an ongoing scientific debate. The "fake debate" strategy was pioneered by the tobacco industry in the 1970s. They used doctors and scientists to reassure the public that smoking didn't cause health problems. These spokespeople were either non-experts or among the small minority of scientists who dissented from the scientific consensus that smoking is bad for you. However, in the public's eye, this conveyed the appearance of ongoing scientific debate. The "Tobacco Strategy" is being used again, but this time to cast doubt on climate science. Less than 3% of climate scientists disagree with the consensus position that humans are causing global warming. However, when the media present the views of a dissenting scientist alongside a mainstream scientist, the public comes away with the mistaken impression of a 50:50 debate. Ironically, the journalistic standard of giving both sides equal weight has ended up distorting the state of climate science. In reality, there is overwhelming agreement among experts that humans are causing global warming. Several surveys of climate scientists have found that 97% agree that humans are causing global warming. Also, over the last 21 years, 97% of scientific papers that state a position on whether humans were causing global warming agree with the consensus. There is overwhelming scientific agreement that humans are driving recent global warming.

MYTH: Recent global warming is caused by the sun, which has been unusually warm.

FACT: A persistent myth is that the Sun is causing global warming.

People persist in this myth by cherry picking data. For example, they look at times in the Earth's past when temperature and solar activity moved in the same direction. But they ignore recent data. In the last few decades of global warming, solar activity and climate have moved in opposite directions. Surface temperatures have increased and global temperatures hit the hottest on record in 2010. At the same time, the Sun has shown a slight cooling trend. In 2009, solar activity reached its lowest levels in over a century. If anything, the drop in solar activity has had a slight cooling influence on climate in recent decades. A number of climate patterns confirm that heat trapping greenhouse gases are causing global warming. Winters are warming faster than summers and nights are warming faster than days. The upper atmosphere is cooling while the lower atmosphere warms. These patterns rule out the sun as a

potential cause of global warming. They also constitute a 'fingerprint' for the fact that humans are causing global warming through greenhouse gas emissions.

Fact only

Thank you for taking part in today's study. The session is almost complete but before you finish, it is important that you read the following debriefing information very carefully—you will be assessed on its contents shortly. Please note that you were exposed to misinformation in this survey. Some of the information in the article "Scientists debate causes of climate change" was in fact misleading and misrepresents the facts. The aim of the current study is to establish whether it is possible to protect members of the general public from deliberately misleading and inaccurate information—known as "misinformation". The following content explains how the previous article misrepresented the facts. Please read the content carefully, as you will be asked questions about it afterwards.
FACT: 97% of climate scientists agree humans are causing global warming.
As empirical evidence for human-caused global warming accumulated, agreement among the scientific community strengthened. A 2009 survey of Earth scientists found that the greater a scientist's expertise in climate science, the more likely they were to endorse the consensus. Among actively publishing climate scientists, they found 97% agreement. The same 97% consensus was found in a 2010 analysis of public statements by climate scientists. An analysis of 21 years of peer-reviewed papers from 1991 to 2011 identified over 4,000 scientific papers that stated a position on whether humans were causing global warming. Among those papers, 97.1% endorsed the consensus. Nearly every reputable, relevant scientific organisation in the world, including the National Academies of Science from 33 different countries, has issued statements endorsing human-caused global warming. There is an overwhelming scientific consensus that humans are causing global warming.
FACT: Climate patterns confirm human-caused global warming.
How do we know that carbon dioxide is the cause of recent global warming? We expect to see a number of distinctive greenhouse patterns in global warming. Observing these patterns strengthens the evidence that humans are causing global warming, as well as eliminating other possible natural causes. Let's have a look at the many human fingerprints on climate change: Greenhouse warming is predicted to cause nights to warm faster than days. This is because at night, the Earth's surface cools by radiating heat out to space. Greenhouse gases trap some of this heat, slowing the night-time cooling. This prediction has been confirmed. Over the last few decades, surface measurements have observed nights warming faster than days. Just as greenhouse gases slow down night-time cooling, they should also slow down winter cooling. Consequently, winters are expected to warm faster than summers. Again, recent analysis of temperature trends over the last few decades bears this out. Both thermometers and satellites find winters warming faster than summers.

Experiment 1 test-phase questions

Myth 1. There is significant disagreement among climate scientists that humans are causing global warming. (1-5 scale from "Strongly disagree" to "Strongly agree).
Myth 2. Recent global warming has been caused by an unusually warm sun. (1-5 scale from "Strongly disagree" to "Strongly agree).
Myth 3. Scientists who disagree that human are causing global warming are a significant proportion of the scientific community. (1-5 scale from "Strongly disagree" to "Strongly agree).

Myth 4. Recent changes in solar activity have had a significant impact on global temperature. (1-5 scale from “Strongly disagree” to “Strongly agree).
Fact 1. An overwhelming majority of climate scientists agree that human activity is causing global warming. (1-5 scale from “Strongly disagree” to “Strongly agree).
Fact 2. Observed patterns in climate change rule out the sun as a major driver of recent climate change. (1-5 scale from “Strongly disagree” to “Strongly agree).
Inference question 1. On a scale from 0 to 100%, how many climate scientists agree that human activity is causing global warming?
Inference question 2. Use the slider to estimate the contribution from human CO2 emissions to increases in temperature: ______ Increase in atmospheric temperature of 0.8 degrees Celsius since 1880

Supplement B

Experiment 2 Experimental Design

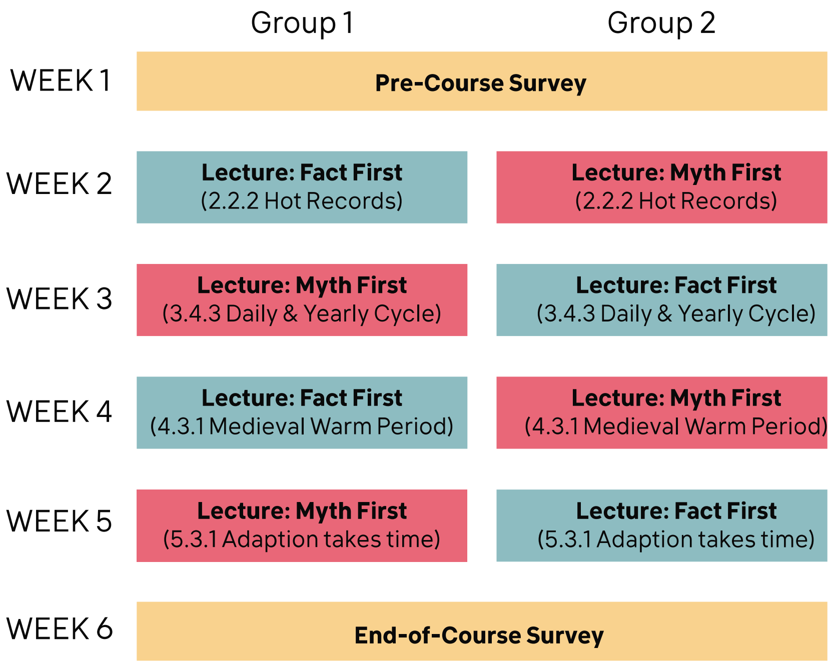

Experiment 2 Climate knowledge items

1. Myth Temperature Records: A record cold winter is indication that global warming is not happening.
2. Fact Medieval Temperatures: Averaged over the planet, temperatures were cooler during medieval times compared to the last few decades.
3. Fact Temperature Records: Recent global warming has increased the number of hot records compared to cold records.
4. Myth Medieval Temperatures: Warm temperatures in medieval times show that recent warming is natural.

5. Myth Species Adaptation: Most species can evolve to adapt to climate change now.
6. Myth Causal Attribution: Recent global warming has been caused by an unusually warm sun.
7. Fact Causal Attribution: Recent global warming has been caused mainly by human activity.
8. Fact Species Adaptation: Species cannot adapt quickly enough to the recent, rapid climate change.

Climate policy items

9. Policy1: Adding a surcharge to electrical bills to establish a fund to help make buildings more energy efficient and teach citizens how to reduce energy use.
10. Policy2: Requiring electric utilities to produce at least 20% of their electricity from renewable energy sources.

11. Policy3: Providing tax rebates for people who purchase energy-efficient vehicles or solar panels.

Climate consensus item

12. On a scale from 0 to 100%, estimate how many climate scientists agree that human activity is causing global warming. Drag the slider below to the appropriate point on the scale.

Descriptive statistics for the full pre-correction sample

For Experiment 4's full pre-correction sample (N = 5291) the average climate knowledge score of the was M = 4.26 (SD = .59) in the standard format and M = 4.27 (SD = .58) in the reverse order format. The climate consensus and climate policy variables were analyzed in order to investigate a possible flow-on effect of the lectures on participant's consensus estimate and policy preferences. In addition to the climate knowledge items, the test survey contained one item that asked participants to estimate the scientific consensus on climate change (0-100%) and three items which measured support for climate policy options discussed in the lectures, also using 5-point Likert scales. The three climate policy items were averaged to form a composite score. The climate consensus estimate was analyzed separately. The total sample out of N = 1002 participants with complete consensus and policy data was N = 966.

Climate consensus

The mean climate consensus score was M = 92.99 (SD = 11.34) for the pre-correction survey and M = 95.79 (SD = 8.98) for the post-correction survey. The Climate Consensus variable appeared non-normally distributed and was unable to be corrected through transformations, however, ANOVA is robust to deviations from normality (Schmider et al., 2010). Non-parametric analyses were comparable and are provided in footnotes. A repeated-measures ANOVA revealed a significant main effect of survey administration stage, $F(1, 965) = 48.20$; $p < .001$; $MSE = 78.30$; $\eta_p^2 = .05$; $BF_{10} = 7.40\text{e+}8$, indicating greater climate consensus after the course.

Climate policy

The mean climate policy score was M = 4.24 (SD = .71) for pre-correction survey and M = 4.37 (SD = .71) for the post-correction survey. A repeated measures ANOVA revealed a significant main effect of survey administration stage, $F(1, 965) = 54.67$; $p < .001$; $MSE = .15$; $\eta_p^2 = .05$; $BF_{10} = 1.48\text{e+}10$, indicating that support for climate policy increase after the course.

Supplement C

Table S1

Claims and their associated brief and detailed explanation

Item	Claim	Explanation	Inference Question	Fact only claim
The Brain Myth - 1	Most people only use between 10 and 50% of their brains.	We use all of our brain: Brain imaging techniques have demonstrated that our whole brain is active, at least to some extent, all of the time. Specific areas of the brain will become more active depending on the demands of the task. The brain, like all other organs, has been shaped by natural selection. It would be extremely costly and inefficient for our body to produce material that it was not going to use.	What percentage of our brain do we use? (0-100%)	People use 100% of their brains.
The Brain Myth - 2	Logical people are left-brained, and creative people are right-brained.	All brains are remarkably similar: A study in 2013 scanned the brains of over 1000 people, and found that all people use both sides of their brain to the same extent, regardless of how creative or logical they were. Some brain functions occur in one side of the brain—for example, language tends to be on the left –yet this is true for virtually everyone.	If your friend was extremely creative, would you assume her brain was structured differently to an individual who was more logical? (0-10 Definitely not - Definitely)	Even though some people are more creative and other people are more logical—people's brains are remarkably similar.
The Brain Myth - 3	Pregnancy affects brain functioning.	Brain functioning is unaffected by pregnancy: A study in the British Journal of Psychiatry assessed the cognitive abilities of over 1000 women, and found no differences between pregnant and non-pregnant women. Many pregnancy guides inform women that they will be abnormally confused or forgetful, so it is possible that normal lapses in memory or thinking are simply misattributed to being pregnant.	If a pregnant friend misplaced her keys, would you attribute it to her being pregnant? (0-10 Definitely not - Definitely)	Brain functioning is unaffected by pregnancy.

Item	Claim	Explanation	Inference Question	Fact only claim
The Brain Fact - 1	Your brain itself feels no pain.	The brain feels no pain: Pain from headaches mostly stems from outside the skull, perhaps from muscle pain in your head or neck or when blood vessels swell and press on nerves. Brain surgery can be performed with only a local anaesthetic to the scalp, as the brain has no pain receptors.	If you have a headache, how likely is it that the pain is stemming from your brain? (0-10 Extremely likely – Extremely unlikely)	Your brain itself feels no pain.
The Brain Fact - 2	The brain is two-thirds fat.	The brain is mostly fat: There are approximately 90 billion neurons in the brain. These neurons are wrapped in myelin, a fat which acts as an electrical insulator. The myelin appears like a string of sausages covering the length of a neuron, allowing the nerve impulses to jump from gap to gap, making the impulses much quicker.	Is the fact that the brain is made up of two thirds fat an accurate way to teach children about biology? (0-10 Definitely not - Definitely)	The brain is two-thirds fat.
The Brain Fact - 3	Brain activity accounts for 20% of our resting metabolic rate.	The brain needs a lot of energy: In 1953, Louis Sokoloff found that the brain consumed the same energy while resting and while doing arithmetic, so the brain uses a lot of energy regardless of what we are doing. Although the brain is only about 2% of the body's total weight, brain activity accounts for 20% of our resting metabolic rate.	If our heart accounts for 10% of our resting metabolic rate, how much does our brain account for? (0-20%)	Brain activity accounts for 20% of our resting metabolic rate.

Item	Claim	Explanation	Inference Question	Fact only claim
Alcohol Myth - 1	If you cook with alcohol, it will 'cook off' and the dish will become non-alcoholic.	Alcohol remains in food: A study in 1992 found that if alcohol was stirred into a dish, 25% of the alcohol remained after a whole hour of cooking. Many people believe that it is easy to cook out the alcohol due to alcohol's low boiling point. Yet even after setting alcohol alight to flambé a dish, a whopping 75% of alcohol remains.	Would you give Christmas pudding flambéed with brandy to a four year old child? (0-10 Definitely not - Definitely)	Dishes with alcohol will remain alcoholic, even after cooking them.
Alcohol Myth - 2	Alcohol promotes sleep.	Alcohol disturbs sleep: Drinking alcohol before bed leads to REM sleep being disrupted. This is followed by abnormally shallow sleep, causing multiple awakenings. The more alcohol consumed prior to sleep, the more pronounced these effects are. So although alcohol may help the onset of sleep, sleep quality is adversely affected.	If your insomniac friend told you they were planning on drinking two glasses of wine before bed to help them sleep, would you advise them otherwise? (0-10 Definitely not - Definitely)	Sleep is adversely affected by alcohol.
Alcohol Myth - 3	Alcohol kills brain cells.	Brain cells survive alcohol: Neurons are the longest living cells in the body, and they are very difficult to kill. Alcoholics and non-alcoholics have the same number and density of neurons in the brain. Alcohol only temporarily affects brain functioning.	Do you think that 'alcohol kills brain cells' is an accurate way to teach high school children about the negative effects of alcohol (0-10 Definitely not - Definitely)	Alcohol negatively impacts brain functioning, but not due to cell death.

Item	Claim	Explanation	Inference Question	Fact only claim
Alcohol Fact - 1	Men and women process alcohol differently.	Alcohol gender differences: ‘Alcohol dehydrogenase’ is an enzyme in the stomach lining which helps break down alcohol. Women possess significantly less of this enzyme than men, which means that even if a man and a woman have equivalent body sizes, the women will feel the effects of alcohol sooner and to a greater extent.	Your friends Ben and Emma are having a drinking competition, shot for shot. They both weigh 60 kilograms. How likely is it that Emma will fall of her chair first? (0-10 Extremely unlikely – Extremely likely)	Men and women process alcohol differently.
Alcohol Fact - 2	Mixing alcohol with sugary drinks makes you more drunk**.**	Diet soda increases the effects of alcohol: A recent study in the journal Alcoholism: Clinical and Experimental Research found that alcohol mixed with diet soda led to participants being more intoxicated than if they consumed the same beverage was mixed with full sugar soda. While participants reported that they did not feel any difference, those drinking diet mixers had significantly slower reaction times and their breathalyser readings were elevated by 18%.	If you wanted to have a few drinks with friends prior to driving, would you choose to mix your drink with diet soda? (0-10 Definitely not - Definitely)	Mixing alcohol with sugary drinks makes you more drunk.
Alcohol Fact - 3	Alcohol improves your sense of smell.	Alcohol can improve sense of smell: The brain automatically dampens our sense of smell, and if these inhibitory signals to the brain are affected, our sense of smell can be enhanced. Consuming alcohol is one example of how these inhibitory signals can be alleviated. A study in the journal of Behavioural Brain Research found that people who had consumed low amounts of alcohol could smell with greater accuracy than those who were sober.	Would a wine expert’s sense of smell improve after drinking the first few tastings? (0-10 Definitely not - Definitely)	Alcohol improves your sense of smell.

Item	Claim	Explanation	Inference Question	Fact only claim
Animals Myth -1	The daddy long legs spider is extremely venomous, but fortunately its fangs are too small to pierce human skin	Daddy long-legs are only mildly venomous: While the fangs of the daddy long legs spider are indeed very small (estimated at 0.25mm in length), they are capable of piercing human skin. Tests have revealed that the venom of the daddy long legs spider is no more powerful than that of most other spiders, and not dangerous to human health.	Scientists believe it is quite likely that the daddy long legs spider will develop larger fangs due to a genetic mutation: do you agree that health officials should fund projects to develop anti-venom? (0 strongly disagree, 10 strongly agree)	While the fangs of the daddy long legs spider can indeed pierce human skin, the venom is only mildly potent.
Animals Myth -2	Urine is an effective treatment for a jellyfish sting.	Urine aggravates jellyfish sting: Urinating on the area affected by a jellyfish sting will only aggravate the sting, not to mention making your day even worse than it already is. While this “home remedy” worked for a character on the television show ‘Friends’, in reality there is no scientific evidence to support it.	Should there be more funding for research isolating which exact properties of urine might be responsible for sting relief? (0-10 Definitely not - Definitely)	Urine will only aggravate a jellyfish sting.
Animals Myth - 3	Bulls are mostly colour-blind, but can see the colour red vividly.	Bulls see mostly blue and yellow: Matadors hold red capes purely due to tradition; it is the movement of the bullfighter’s cape that causes the bull to charge. Bulls can see colour, but they only have two types of cones in their eyes, rather than three types like us humans. This means that they can see blues and yellows, but ironically, not reds.	What is the likelihood that a bull would charge at a matador brandishing a blue cape? (0: Extremely unlikely – 10 Extremely likely)	Bulls can see blues and greens, but not reds.

Item	Claim	Explanation	Inference Question	Fact only claim
Animals Fact -1	The colour of a chicken’s egg is related to the chicken’s ear lobe.	Egg colour is related to chicken’s earlobes: Chickens’ earlobes are quite large, yet are covered with feathers so are rarely visible. The earlobes do not have a direct impact upon the colour of the egg, but reflect trends in genetics, like people with black hair being more likely to have brown eyes. Chicken breeds with white earlobes are likely to lay white eggs, and chickens with red earlobes are likely to lay brown eggs.	If you bought a box of white eggs, what is the likelihood that the chickens that laid them have white earlobes? (0: Extremely unlikely – 10 Extremely likely)	The colour of a chicken’s egg is related to the chicken’s ear lobe.
Animals Fact - 2	Dogs can smell cancer.	Dogs smell cancer: Cancer patients have traces of chemicals (like alkanes and benzene derivatives) in their breath, and dogs can detect chemicals in concentrations as small as a few parts per trillion. The University of California did the first double blind study, where dogs correctly detected 99% of lung cancer samples, and made a mistake with only 1% of healthy controls.	How much extra funding should go towards programs supporting training for cancer sniffing dogs? (0-100%)	Dogs can smell cancer.
Animals Fact - 3	A cockroach can live for over a week without its head	Cockroaches survive headless: A cockroach’s body works very differently to that of a human, which allows it to survive after it has been decapitated. A cockroach does not breathe through its mouth, but through ‘spiracles’, which are little holes in its body segment. Additionally, its nervous system is located throughout their body, so they do not need their brain to control all bodily functions. Lastly, they need much less food than a human.	What proportion of cockroaches live after they have been decapitated? (0-100%)	A cockroach can live for over a week without its head

Item	Claim	Explanation	Inference Question	Fact only claim
Flu vaccine Myth -1	The side effects of the flu vaccine are worse than the flu.	Flu is worse than vaccine side effects: The worst side effect you're likely to get with injectable vaccine is a sore arm. The nasal mist flu vaccine might cause nasal congestion, runny nose, sore throat and cough. The risk of a rare allergic reaction is far less than the risk of severe complications from influenza.	Would concerns regarding the side effects of the flu vaccine be sufficient to deter you from getting vaccinated?<br>(0-10 Definitely not - Definitely)	Flu is much worse than any side effects of the flu vaccine.
Flu vaccine Myth - 2	Only older people need the flu vaccine.	Flu vaccine is good for all ages: Adults and children with conditions like asthma, diabetes, heart disease, and kidney disease need to get flu vaccine. People who are active and healthy can benefit from the protection the flu vaccine offers. The flu vaccine is beneficial to all age groups.	How likely is it that you will get a flu vaccine in the future?<br>(0: Extremely unlikely – 10 Extremely likely)	The flu vaccine is beneficial to all age groups, not only the elderly.
Flu vaccine Myth - 3	You must get a flu vaccine before winter for it to be effective.	Flu vaccine still effective in winter: Flu vaccine can be given before or during the flu season. It only takes about two weeks for antibodies to develop in the body, so as long as the current season’s flu is in circulation, the vaccine will be beneficial. While the best time to get flu vaccine is in autumn, getting immunized in winter can still protect you against the flu.	If it was winter, would you be less inclined to get a flu vaccine than in autumn?<br>(0-10 Definitely not - Definitely)	If you receive the flu vaccination in winter, you will still be protected.

Item	Claim	Explanation	Inference Question	Fact only claim
Flu vaccine Fact - 1	Not everyone can take the flu vaccine	Some people should avoid flu vaccine: You might not be able to get the flu vaccine if you are allergic to eggs (used in making the vaccine), are very ill with a high fever, or have had a severe reaction to the flu vaccine in the past. Other rare conditions such as Guillain-Barre syndrome, an uncommon paralysing illness, may also prevent you from receiving the flu vaccine.	Would you ask your friend about their allergies before recommending the flu vaccine to them?<br>(0-10 Definitely not - Definitely)	Not everyone can take the flu vaccine
Flu vaccine Fact - 2	Even if I get the flu vaccine, I can still get a mild case of the flu.	Mild flu and the vaccine: The flu vaccine protects most people from the flu. Sometimes a vaccinated person will still get the flu, but they will be far less sick than without the vaccine. Flu vaccines will not protect you 100% from mild cases of the flu.	How likely is it that you would encourage your own friends or relatives to get a flu vaccine?<br>(0-10 Extremely unlikely – extremely likely)	Even if I get the flu vaccine, I can still get a mild case of the flu.
Flu vaccine Fact - 3	People can die from the flu.	The flu can kill: Influenza (flu) is a highly infectious disease of the lungs, and it can lead to pneumonia. Each year about 114,000 people in the United States are hospitalized and about 36,000 people die because of the flu. Most who die are 65 years and older. But small children less than 2 years old are as likely as those over 65 to have to go to the hospital because of the flu.	How would you rate the potential benefits of flu vaccination?<br>(0-10)	People can die from the flu.

Item	Claim	Explanation	Inference Question	Fact only claim
Hypnosis Myth -1	A talented hypnotist can hypnotise you against your will.	Hypnosis is voluntary: Hollywood movies and dramatized hypnosis performances have distorted people's view of hypnosis. During hypnosis, individuals remain fully aware of their behaviour. They can easily refuse to comply with the hypnotist's suggestions if they wish to do so.	How likely is it that a talented hypnotist could make you jump off a cliff? (0: Extremely unlikely – 10 Extremely likely)	It is impossible for any hypnotist, no matter how talented they are, to hypnotise you against your will.
Hypnosis Myth -2	Hypnosis is useful for retrieving repressed memories.	Memories "recovered" using hypnosis usually false: While it is plausible that an individual could suddenly recall a traumatic event they had repressed, the memory is more likely to be valid if it occurs spontaneously, rather than working towards a revelation in therapy or during hypnosis. Memories recovered during hypnosis are more likely to be false memories resulting from suggestion.	If your friend tells you about a memory that was helped to be uncovered by a hypnotherapist, would you assume that it is a true event? (0: Extremely unlikely – 10 Extremely likely)	Repressed memories uncovered during hypnosis are likely to be false memories.
Hypnosis Myth - 3	Under hypnosis, a person can perform better physically and mentally.	Hypnosis unrelated to performance: Hypnosis is likely to result from a mixture of relaxation, conformity, obedience, suggestion and role-playing. Some studies find that participants perform worse while under hypnosis, as participants make more errors, and show less motivation to do well. Other studies find no performance differences in intellectual or physical performance.	Do you think you could do a crossword puzzle quicker and more accurately if you were hypnotised, than under normal circumstances? (0-10 Definitely not - Definitely)	A person's abilities will be approximately the same, whether or not they are hypnotised.

Item	Claim	Explanation	Inference Question	Fact only claim
Hypnosis Fact -1	Hypnosis can be used in pain management	Hypnosis reduces pain: People tend to have a higher pain threshold whilst hypnotised. It has been performed during childbirth and other medical procedures such as the dressing change of burn patients, and seems to help patients manage their pain.	If you were in a great deal of pain, and hypnosis was offered, would you accept? (0-10 Definitely not - Definitely)	Hypnosis can be used in pain management
Hypnosis Fact - 2	It is possible to ‘hypnotise’ animals.	Animals can be hypnotised: In animals, hypnotic states are commonly referred to as ‘tonic immobility.’ It is thought to be an involuntary reflex that an animal enters in response to a threat. For example, many species of shark become immobile if you turn them upside down, and lobsters can be put into this state if you stroke their head. It is possible to hypnotise an animal as it plays a role in their survival, helping the animal not be noticed, or ‘play dead’.	If we gave you an animal, and instructions on how to hypnotise it, what is the likelihood that it would work? (0: Extremely unlikely – 10 Extremely likely)	It is possible to ‘hypnotise’ animals.
Hypnosis Fact - 3	The susceptibility to be hypnotised varies from person to person.	Individual differences in hypnosis: There are scales such as the ‘Stanford Hypnotic Susceptibility Scale’, which measure an individual’s susceptibility to hypnosis. Your motivations (how much you want to be hypnotised), and expectations (how much you believe you will be hypnotised) influence the likelihood that you will be hypnotised.	If your friend really wanted to be hypnotised, but you did not – would the hypnotist be able to hypnotise both of you? (0-10 Definitely not - Definitely)	The susceptibility to be hypnotised varies from person to person.

Table S2

Planned Comparisons on Fact Belief Ratings in Experiment 3

	Standard	Reverse order	Facts-only	Myths-only
Reverse order	F = 0.34<br>p = .56<br>BF_{01} = 5.57			
Facts-only	F = 0.01<br>p = .93<br>BF_{01} = 6.41			
Myths-only	F = 101.88<br>p < .001*<br>BF_{10} = 1.47e+20			
Control	F = 85.17<br>p < .001*<br>BF_{10} = 2.02e+16	F = 79.87<br>p < .001*<br>BF_{10} = 3.38e+15	F = 83.85<br>p < .001*<br>BF_{10} = 4.47e+15	F = 0.55<br>p = .46<br>BF_{01} = 4.89

Note. All df1 = 1, df2 = 98; * indicates significance after Holm-Bonferroni correction.

Table S3

Planned Comparisons on Fact Inference Scores in Experiment 3

	Standard	Reverse order	Facts-only	Myths-only
Reverse order	F = 0.55<br>p = .46<br>BF_{01} = 5.04			
Facts-only	F = 0.03<br>p = .86<br>BF_{01} = 6.25			
Myths-only	F = 95.49<br>p < .001*<br>BF_{10} =<br>1.21e+17			
Control	F = 114.11<br>p < .001*<br>BF_{10} =<br>1.21e+21	F = 101.59<br>p < .001*<br>BF_{10} =<br>6.59e+18	F = 95.02<br>p < .001*<br>BF_{10} =<br>1.13e+20	F = 2.45<br>p = .12<br>BF_{01} = 1.64

Note. All df1 = 1, df2 = 98; * indicates significance after Holm-Bonferroni correction.

Table S4

Planned Comparisons on Fact Belief Ratings in Experiment 4

	Standard	Reverse order	Facts-only	Myths-only
Reverse order	F = .46<br>p = .500<br>BF_{01} = 7.43			
Facts-only	F = .32<br>p = .571<br>BF_{01} = 7.70			
Myths-only	F = 385.72<br>p < .001*<br>BF_{10} = 1.04e+54			
Control	F = 164.34<br>p < .001*<br>BF_{10} = 1.28e+32	F = 168.53<br>p < .001*<br>BF_{10} = 9.19e+29	F = 196.56<br>p < .001*<br>BF_{10} = 2.48e+34	F = 14.58<br>p < .001*<br>BF_{10} = 271.34

Note. All df1 = 1, df2 = 195; * indicates significance after Holm-Bonferroni correction.

Table S5

Planned Comparisons on Fact Inference Scores in Experiment 4

	Standard	Reverse order	Facts-only	Myths-only
Reverse order	F = 2.17<br>p = .142<br>BF_{01} = 3.27			
Facts-only	F = .49<br>p = .483<br>BF_{01} = 7.42			
Myths-only	F = 91.86<br>p < .001*<br>BF_{10} = 1.41e+19			
Control	F = 71.96<br>p < .001*<br>BF_{10} = 1.28e+15	F = 60.73<br>p < .001*<br>BF_{10} = 2.12e+11	F = 55.12<br>p = .001*<br>BF_{10} = 3.89e+12	F = 2.26<br>p = .134<br>BF_{01} = 2.71

Note. All df1 = 1, df2 = 195; * indicates significance after Holm-Bonferroni correction.

Analysis Excluding Participants who did not Receive the Alcohol Topic

Experiment 2 results were also run excluding participants (n = 36) who did not receive the "alcohol" topic in the standard format condition. The final sample was N = 161. Results were comparable to earlier analyses.

Belief scores

Mean belief scores across conditions are shown in Figure S1. By and large, explanations were again affective, and explanation format again made very little difference to the belief change achieved. Retention interval had the expected effect: Participants were slightly more likely to correctly label myths as false and facts as true after one week than after three weeks.

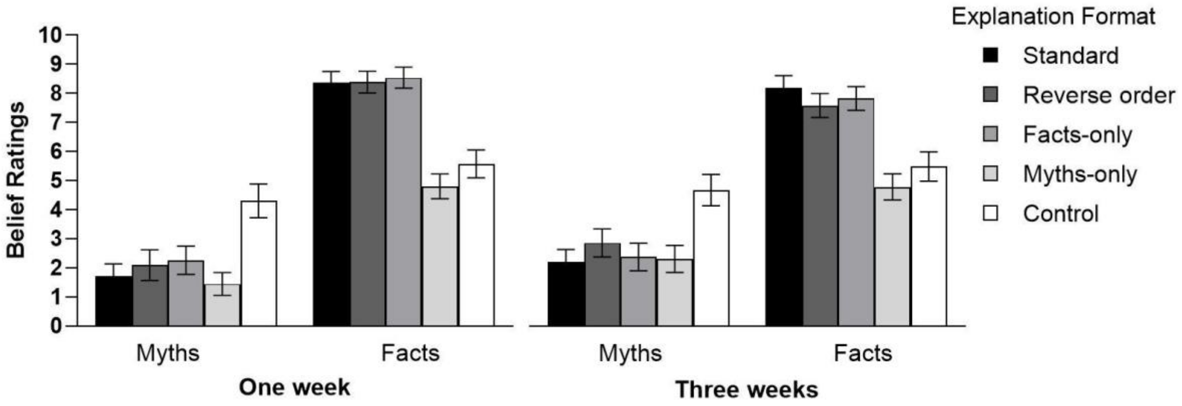

Figure S1. Belief ratings across conditions in Experiment 2. Error bars denote 95% confidence intervals.

A 2 × 5 within-between ANOVA with factors retention interval (one week vs. three weeks) and explanation format (standard vs. reverse vs. facts-only vs. myths-only vs. control) was performed on the myth belief scores, revealing two main effects. The main effect of retention interval, $F(1, 159) = 5.87$; $p = .017$; $MSE = 9.33$; $\eta_p^2 = .04$; $BF_{10} = 1.61$, indicated that myth belief increased between one and three weeks. The main effect of explanation format, $F(3.66, 581.48) = 50.83$; $p < .001$; $MSE = 3.99$; $\eta_p^2 = .24$; $BF_{10} = 1.47e+34$, indicated that belief ratings differed across explanation formats, meaning that all explanation formats led to sustained belief change relative to the control condition.

Planned comparisons, applying Holm-Bonferroni correction, are presented in Tables S6 and 7 for myths and facts, respectively. To limit the number of comparisons, we collapsed over retention-interval conditions, which did not seem to differ systematically. For myths, results confirmed that (1) all retraction formats differed from control, and that (2) the standard format had greater efficacy compared to the reverse order format, but not the facts-only and myths-only formats.

For facts, again all affirmation formats differed from control. The standard format did not differ from the other affirmation formats, with the exception of the myths-only condition, which did not actually feature fact affirmations, and indeed produced even *lower* scores than control. As participants were not presented with any affirmations in this condition, they seemingly assumed that any information presented regarding the relevant topic was false.

Inference scores

Mean inference scores are provided in Figure S2. First, a 2 × 5 within-between ANOVA with factors retention interval and explanation format was performed on participants' mean myth inference scores. There was a main effect of retention interval, $F(1,159) = 4.41$; $p = .037$; $MSE = 5.55$; $\eta_p^2 = .03$; $BF_{10} = .734$, indicating that belief tended to increase slightly over time, and a main effect of explanation format, $F(4, 636) = 29.29$; $p < .001$; $MSE = 2.98$; $\eta_p^2 = .16$; $BF_{10} = 4.692e+19$, indicating that corrections reduced inference scores.

Analogous to belief ratings, planned comparisons were performed on myth inference scores (see Table S8). All correction formats differed from control in the expected direction, and the standard format had lower inference scores than the reverse order format, but not the facts-only and myths-only correction formats.

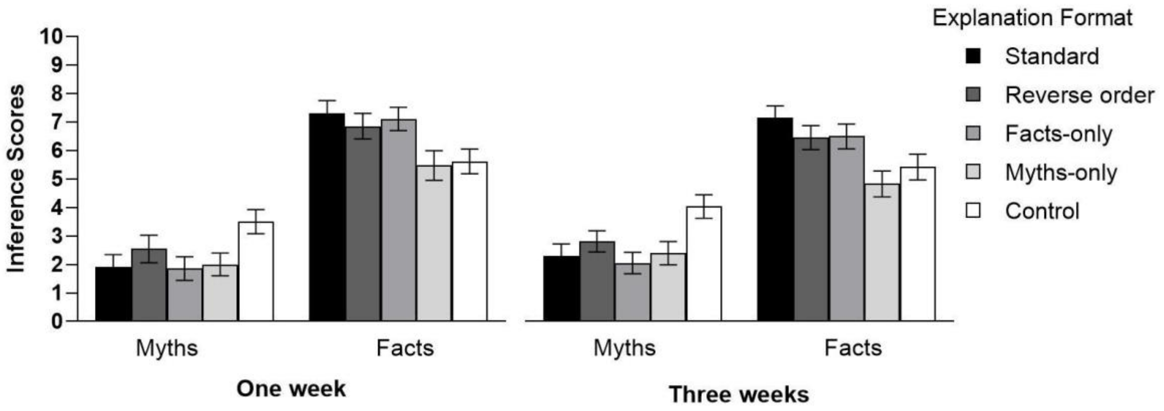

Figure S2. Inference scores across conditions in Experiment 2. Error bars denote 95% confidence intervals.

Next, a 2 × 5 within-between ANOVA was performed on fact inference scores. There was a main effect of retention interval, $F(1,159) = 8.14$; $p = .005$; $MSE = 4.05$; $\eta_p^2 = .05$; $BF_{10} = 1.288$, indicating that fact belief decreased between one and three weeks, and a main effect of explanation format, $F(3.57, 568.14) = 31.09$; $p < .001$; $MSE = 4.57$; $\eta_p^2 = .17$; $BF_{10} = 1.701e+22$, indicating that affirmations were associated with greater scores.

Planned comparisons are shown in Table S8 and S9. All conditions differed from control, apart from the myths-only format (which featured no factual affirmations). Additionally, the reverse order format was less effective than the standard format.

Table S6

Planned Comparisons on Myth Belief Ratings in Experiment 4 Replication

	Standard	Reverse order	Facts-only	Myths-only
Reverse order	*F* = 6.46<br>*p* = .012*<br>BF_{10} = 2.59			
Facts-only	*F* = 3.85<br>*p* = .052<br>BF_{01} = 1.27			
Myths-only	*F* = 0.18<br>*p* = .671<br>BF_{01} = 7.66			
Control	*F* = 111.58<br>*p* < .001*<br>BF_{10} = 1.20e+20	*F* = 65.94<br>*p* < .001*<br>BF_{10} = 7.31e+11	*F* = 76.99<br>*p* < .001*<br>BF_{10} = 1.35e+14	*F* = 128.97<br>*p* < .001*<br>BF_{10} = 4.78e+21

Note. All df1 = 1, df2 = 159; * indicates significance after Holm-Bonferroni correction.

Table S7

Planned Comparisons on Fact Belief Ratings in Experiment 4 Replication

	Standard	Reverse order	Facts-only	Myths-only
Reverse order	*F* = 2.86<br>*p* = .093<br>BF_{01} = 2.11			
Facts-only	*F* = .44<br>*p* = .507<br>BF_{01} = 6.66			
Myths-only	*F* = 356.27<br>*p* < .001*<br>BF_{10} = 2.25e+47			
Control	*F* = 138.65<br>*p* < .001*<br>BF_{10} = 2.46e+27	*F* = 125.59<br>*p* < .001*<br>BF_{10} = 6.68e+22	*F* = 143.93<br>*p* < .001*<br>BF_{10} = 1.98e+25	*F* = 9.93<br>*p* = .002*<br>BF_{10} = 26.53

Note. All df1 = 1, df2 = 159; * indicates significance after Holm-Bonferroni correction.

Table S8

Planned Comparisons on Myth Inference Scores in Experiment 4 Replication

	Standard	Reverse order	Facts-only	Myths-only
Reverse order	F = 7.61<br>p = .006*<br>BF_{10} = 6.01			
Facts-only	F = .85<br>p = .359<br>BF_{01} = 5.50			
Myths-only	F = .21<br>p = .650<br>BF_{01} = 7.54			
Control	F = 79.03<br>p <.001*<br>BF_{10} = 3.68e+13	F = 25.32<br>p <.001*<br>BF_{10} = 76026	F = 99.54<br>p <.001*<br>BF_{10} = 5.32e+16	F = 68.21<br>p <.001*<br>BF_{10} = 1.72e+12

Note. All df1 = 1, df2 = 159; * indicates significance after Holm-Bonferroni correction.

Table S9

Planned Comparisons on Fact Inference Scores in Experiment 4 Replication

	Standard	Reverse order	Facts-only	Myths-only
Reverse order	F = 7.92<br>p = .006*<br>BF_{10} = 5.91			
Facts-only	F = 4.81<br>p = .030<br>BF_{10} = 1.17			
Myths-only	F = 72.22<br>p < .001*<br>BF_{10} = 9.79e+14			
Control	F = 54.03<br>p < .001*<br>BF_{10} = 3.40e+11	F = 31.83<br>p < .001*<br>BF_{10} = 399247	F = 28.46<br>p = .001*<br>BF_{10} = 3.170e+6	F = 2.29<br>p = .132<br>BF_{01} = 2.351

Note. All df1 = 1, df2 = 159; * indicates significance after Holm-Bonferroni correction.
